# Low Spatial Peak-to-Average Power Ratio Transmission for Improved Energy Efficiency in Massive MIMO Systems

**DOI:** 10.3390/s21165534

**Published:** 2021-08-17

**Authors:** Sina Rezaei Aghdam, Thomas Eriksson

**Affiliations:** Department of Electrical Engineering, Chalmers University of Technology, 412 96 Gothenburg, Sweden; thomase@chalmers.se

**Keywords:** energy efficiency, massive MIMO, downlink, linear precoding, spatial PAPR, nonlinear power amplifier

## Abstract

A significant portion of the operating power of a base station is consumed by power amplifiers (PAs). Much of this power is dissipated in the form of heat, as the overall efficiency of currently deployed PAs is typically very low. This is because the structure of conventional precoding techniques typically results in a relatively high variation in output power at different antennas in the array, and many PAs are operated well below saturation to avoid distortion of the transmitted signals. In this work, we use a realistic model for power consumption in PAs and study the impact of power variation across antennas in the array on the energy efficiency of a massive MIMO downlink system. We introduce a family of linear precoding matrices that allow us to control the spatial peak-to-average power ratio by projecting a fraction of the transmitted power onto the null space of the channel. These precoding matrices preserve the structure of conventional precoders; e.g., they suppress multiuser interference when used together with zeroforcing precoding and bring advantages over these precoders by operating PAs in a more power-efficient region and reducing the total radiated distortion. Our numerical results show that by controlling the power variations between antennas in the array and incorporating the nonlinearity properties of PA into the precoder optimization, significant gains in energy efficiency can be achieved over conventional precoding techniques.

## 1. Introduction

Massive multiple-input-multiple-output (MIMO) has become the key technology to meet the capacity requirements of fifth-generation (5G) wireless communications and beyond [1]. The basic idea of massive MIMO is to equip the base station with many antennas and serve multiple users over the same time/frequency resources through spatial multiplexing. However, the performance benefits of massive MIMO come from using more hardware, e.g., multiple radio frequency (RF) chains per base station. Therefore, the study and optimization of the total cost and energy efficiency of massive MIMO systems has been an active research area over the last decade [2].

In this context, energy efficiency is typically defined as the number of bits that can be reliably transmitted per unit of power consumed. The authors in [3] consider a model to quantify the power consumed by digital signal processing and analog circuits in a multi-user MIMO system. This model is then used for analyses that provide insight into how to choose the number of antennas at the base station, the number of user equipment (UEs) served, and the transmit power to maximize energy efficiency. A key finding of [3] is that energy-efficient multi-user systems operate in a high signal-to-noise ratio (SNR) region where interference-suppressing processing, such as zeroforcing (ZF) precoding, is strongly preferable to interference-ignoring transmission schemes, such as maximum-ratio transmission (MRT).

A significant portion of the operating energy of a base station is consumed by the power amplifiers (PAs); see, e.g., Reference [4]. Much of this power is dissipated as heat, as the overall efficiency of currently deployed PAs is typically very low. This is because in conventional transmission schemes, many of the PAs are operated well below saturation to avoid distortion of the transmitted signals. Therefore, a reasonable approach to improve energy efficiency is to design the transmission schemes in such a way that the PA power dissipation is minimized. Examples of such energy efficient transmission schemes are proposed in [5,6,7]. The authors in [5] consider a single-user MIMO channel and propose a transmission power allocation strategy that provides an improved rate for a given consumed power compared to conventional MRT transmission. In [6], it is shown that transmit antenna selection can achieve near-optimal performance (in terms of ergodic capacity) over a multiple-input-single-input (MISO) channel under constraints on per-antenna and total power consumed. More recently, the authors in [7] extended the analysis of [5,6] to point-to-point and multiuser MIMO scenarios. In the multiuser case, a non-convex optimization problem is formulated and approached to minimize the total power consumed at the PAs such that a certain minimum signal-to-interference-plus-noise ratio (SINR) is achieved by all UEs.

The solutions in [5,6,7] do not take into account the effects of the distortion introduced by the nonlinear PAs on the quality of the received signal. The amount and directivity of the nonlinear distortion depend strongly on the covariance matrix of the beamformed signal [8,9]. Motivated by this fact, a precoder optimization framework was proposed in [10] that incorporates the PA nonlinearity properties in maximizing the spectral and energy efficiency.

As a continuation of the work in [10], in this paper, we study the impact of per-antenna power distribution among different antennas in the array on the energy efficiency of a massive MIMO downlink system. We show that using a precoding matrix with a lower spatial peak-to-average power ratio (SPAPR), it is possible to operate all PAs with high power efficiency. Moreover, with such a precoding matrix, the total amount of distortion emanating from nonlinear PAs can be drastically reduced by using a relatively small backoff. We introduce a family of linear precoding matrices generated by adding a term to the expression of conventional precoding matrices and exploiting the excess of spatial degrees of freedom in massive MIMO systems. This term is projected onto the null space of the channel and adjusted such that the resulting precoding matrix provides equal transmit power at different antennas. The beneficial properties of this precoding scheme are achieved at the cost of increased total transmission power and by wasting some of this power by transmitting into null space. Accordingly, transmission with zero power variation can be significantly suboptimal in different scenarios. For this reason, we introduce a framework that allows a more flexible control of SPAPR by jointly optimizing both the precoder matrix and the power variation across the antennas in the array. Finally, we perform numerical simulations to compare the energy efficiency of the proposed precoding solutions with that of conventional ZF precoding.

The remainder of this paper is organized as follows. In Section 2, we introduce the system model considered and give relevant formulations for quantifying the spectral and energetic efficiency. The impact of power variations between antennas in the array on the spectral and energy efficiency is studied in Section 3. In Section 4, a simple framework for generating precoding matrices with equal antenna powers is presented. This framework is extended to a more general case with flexible control of SPAPR in Section 5. Numerical results are given in Section 6, and finally the paper is concluded in Section 7.

*Notation:* The vectors and matrices are marked in bold with lower and upper case letters. The superscripts ${\left(\cdot \right)}^{*}$, ${\left(\cdot \right)}^{T}$, and ${\left(\cdot \right)}^{H}$ denote the complex conjugate, the transpose, and the Hermitian transpose, respectively. We use E[·] to denote the expected value. Moreover, ∥a∥ is used to denote the $\ell_{2}$-norm of the vector a. The M×M identity matrix is denoted by $I_{M}$. By A⊙B, we denote the Hadamard product (entry-wise product) of two equally sized matrices A and B. Furthermore, diag(a) represents a diagonal matrix containing the elements of the vector a on its diagonal, and diag(A) is the principal diagonal of a square matrix A. The element-wise magnitude of a matrix A is represented by |A|. The distribution of a circularly symmetric complex Gaussian random vector with covariance matrix $C\in C^{M\times M}$ is denoted by CN(0,C).

## 2. System Model and Preliminaries

**Figure 1:**
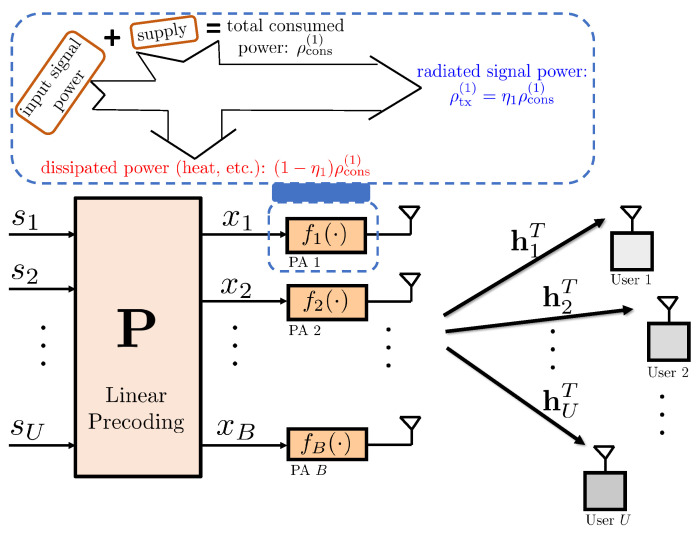
System model: multiuser MIMO downlink with single-antenna UEs. The base station is equipped with *B* transmit antennas serving *U* UEs via spatial multiplexing. Each transmit antenna is equipped with a PA whose nonlinearity characteristics and power consumption are modeled as (2) and (20), respectively.

We consider the downlink of a multiuser MIMO transmission where the base station uses a co-located array of *B* antennas to serve *U* single-antenna UEs as depicted in Figure 1. The data symbols to be transmitted to the UEs, $s=\left[s_{1},s_{2},\cdots ,s_{U}\right]∼\mathrm{CN}\left(0,I_{U}\right)$, are precoded using a linear precoding matrix $P=\left[p_{1},\cdots ,p_{U}\right]\in C^{B\times U}$, yielding the precoded signal

(1) $$x=[x_{1},x_{2},\cdots ,x_{B}]=Ps.$$

The signal at each antenna branch $x_{b}$ passes through a nonlinear PA. The nonlinear characteristics of the PAs are modeled using (2K+1)th order polynomial as [11]

(2) $$f_{b}\left(x_{b}\right)=\sum_{k=0}^{K}\beta_{2k+1}^{\left(b\right)}x_{b}|x_{b}{|}^{2k}=\beta_{1}^{\left(b\right)}x_{b}+\beta_{3}^{\left(b\right)}x_{b}|x_{b}{|}^{2}+\cdots +\beta_{2K+1}^{\left(b\right)}x_{b}{\left|x_{b}\right|}^{2K},$$

where $\beta_{1}^{\left(b\right)},\beta_{3}^{\left(b\right)},\cdots ,\beta_{2K+1}^{\left(b\right)}$ are complex-valued model parameters corresponding to the *b*th PA capturing both amplitude-to-amplitude modulation (AM/AM) and amplitude-to-phase modulation (AM/PM) distortions.

Considering the multiuser MIMO system model in Figure 1, the received signal at the *u*th user is given by

(3) $$y_{u}=h_{u}^{T}f\left(x\right)+w_{u},$$

where $h_{u}\in C^{B}$ is the channel vector, x is the precoded signal as in (1), and $f\left(x\right)={\left[f_{1}\left(x_{1}\right),\cdots ,f_{B}\left(x_{B}\right)\right]}^{T}$ denotes the output of the PAs as described in (2). Furthermore, $w_{u}∼\mathrm{CN}\left(0,N_{0}\right)$ is the additive white Gaussian noise (AWGN).

### 2.1. Channel Model

In this paper, we use a channel model that captures the sparse scattering properties of millimeter-wave channels in non-line-of-sight (nLoS) environments, namely when there is no dominant path. In this model, typically referred to as a geometric channel model [12], each scatterer contributes to a single path, and the channel coefficients can be expressed as follows:

(4) $$h_{u}=\sqrt{\frac{B}{N_{\mathrm{path}}}}\sum_{\ell =1}^{N_{\mathrm{path}}}\zeta_{u,\ell }a\left(\psi_{u,\ell }\right),$$

for u=1,⋯,U, where $N_{\mathrm{path}}$ represents the number of paths. Moreover, $\psi_{u,\ell }$ is the angle of departure (AoD) for the *ℓ*th path, and $a(\psi_{u,\ell })$ is the corresponding array response vector. For a uniform linear array (ULA) with half a wavelength antenna element separation, the *b*th entry of the array response vector is given by

(5) $${\left[a(\psi_{u,\ell })\right]}_{b}=\frac{1}{\sqrt{B}}e^{-j\pi \left(b-1\right)\cos\left(\psi_{u,\ell }\right)},$$

for b=1,⋯,B. Furthermore, $\zeta_{u,\ell }∼\mathrm{CN}\left(0,\gamma_{u}^{2}\right)$ is the independent and identically distributed (i.i.d.) channel gain (including path loss) corresponding to the *ℓ*th path. Throughout the paper, we assume that the UEs know the channel coefficients perfectly. However, on the transmitter side, we consider the availability of both the perfect and imperfect channel state information (CSI).

### 2.2. A Lower Bound on the Sum Rate Capacity

The nonlinear power amplifiers introduce distortion into the transmitted signal, which can significantly degrade the capacity of the multiuser MIMO system. As a first step in deriving a tractable approximation of the ergodic sum rate capacity of the channel input-output model in (3), we use Bussgang’s theorem [13], which allows us to decompose the output of the nonlinear function into a scaled linear signal and an uncorrelated distortion. Since x is circularly symmetric complex Gaussian distribution (which follows from the assumption of Gaussian s), we can decompose f(x) into

(6) f(x)=Gx+e,

where $G=\mathrm{diag}\left(g_{1},g_{2},\cdots ,g_{B}\right)$ is diagonal matrix whose entries are the Bussgang gain values given by [14]

(7) $$g_{b}=\frac{𝔼\left[f\left(x_{b}\right)x_{b}^{*}\right]}{𝔼\left[|x_{b}{|}^{2}\right]}=\frac{\sum_{k=0}^{K}\beta_{2k+1}^{\left(b\right)}𝔼\left[|x_{b}{|}^{2k+2}\right]}{𝔼\left[|x_{b}{|}^{2}\right]}.$$

The distortion term in (6), i.e., $e\in C^{B}$, is uncorrelated with x, i.e., $E\left[xe^{H}\right]=0_{B\times B}$. For the (2K+1)th order polynomial model in (2), the Bussgang gain in (7) can be derived using the moments of complex Gaussian random variables [15] as

(8) $$g_{b}=\frac{\sum_{k=0}^{K}\beta_{2k+1}^{\left(b\right)}\left(k+1\right)!\;{\left(\sigma_{x_{b}}^{2}\right)}^{k+1}}{\sigma_{x_{b}}^{2}}=\sum_{k=0}^{K}\left(k+1\right)!\;\beta_{2k+1}^{\left(b\right)}{\left(\sigma_{x_{b}}^{2}\right)}^{k},$$

where $\sigma_{x_{b}}^{2}$ is the variance of the precoded signal at the *b*th antenna, i.e., $x_{b}$. Using (1), the matrix G can be derived as a function of the precoding matrix P as

(9) $$\begin{array}{cc} G(P) & =\sum_{k=0}^{K}\left(k+1\right)!\;A_{2k+1}\mathrm{diag}{\left(C_{x}\right)}^{k} \end{array}$$

(10) $$\begin{array}{cc} \; & =A_{1}I_{B}+2A_{3}\mathrm{diag}\left(C_{x}\right)+\cdots +\left(K+1\right)!\;A_{2K+1}\mathrm{diag}{\left(C_{x}\right)}^{K}, \end{array}$$

where

(11) $$A_{2k+1}=\mathrm{diag}\left(\left[\beta_{2k+1}^{\left(1\right)},\cdots ,\beta_{2k+1}^{\left(B\right)}\right]\right),$$

for k=1,⋯,K and $C_{x}=E\left[xx^{H}\right]=PP^{H}$ is the input covariance matrix. Using (9) and following a similar approach as in, e.g., Equation (11) of Reference [8], the covariance matrix of the distortion e can be derived as

(12) $$C_{e}\left(P\right)=\sum_{k=1}^{K}L_{k}C_{x}⊙{\left|C_{x}\right|}^{2k}L_{k}^{H},$$

where

(13) Lk=1k+1∑l=kKlk(l+1)!A2l+1diag(Cx)l−k.

The linear decomposition described in (6)–(13) allows for derivation of a lower bound on the ergodic sum rate capacity. Substituting the Bussgang decomposed transmitted signal in (3) yields

(14) $$\begin{array}{c} y_{u}=h_{k}^{T}G\left(P\right)Ps+h_{k}^{T}e+w_{k}=h_{u}^{T}G\left(P\right)p_{u}s_{u}+\underset{w_{\mathrm{eff},u}}{\underbrace{\sum_{r\neq u}h_{u}^{T}G\left(P\right)p_{r}s_{r}+h_{u}^{T}e+w_{u}}}, \end{array}$$

where $w_{\mathrm{eff},u}$ is the effective noise that consists of the inter-user interference, the received nonlinear distortion, and the AWGN terms. In view of the fact that $w_{\mathrm{eff},u}$ is not Gaussian, the exact evaluation of the capacity is not straightforward. Using the so-called “auxiliary channel lower bound” [16] and via replacing $w_{\mathrm{eff},u}$ by a complex Gaussian noise ${\tilde{w}}_{\mathrm{eff},u}$ that has the same variance as $w_{\mathrm{eff},u}$, the following achievable sum rate is obtained in closed form

(15) $$R_{\mathrm{sum}}\left(P\right)=\sum_{u=1}^{U}\log_{2}\left(1+{\mathrm{SIN}\mathrm{DR}}_{u}\left(P\right)\right),$$

where ${\mathrm{SIN}\mathrm{DR}}_{u}\left(P\right)$ denotes the signal-to-interference-noise-and-distortion ratio (SINDR) at the *u*th user and is given as

(16) $$\begin{array}{c} {\mathrm{SIN}\mathrm{DR}}_{u}\left(P\right)=\frac{|h_{u}^{T}G\left(P\right)p_{u}{|}^{2}}{\sum_{r\neq u}{\left|h_{u}^{T}G\left(P\right)p_{r}\right|}^{2}+h_{u}^{T}C_{e}\left(P\right)h_{u}^{*}+N_{0}}. \end{array}$$

It is worth noting that the lower bound in (15) and (16) corresponds to the ergodic sum rate that can be obtained using a Gaussian codebook and a mismatched nearest-neighbor decoder at the UEs under the assumption that the channel coefficients $h_{u}$ are perfectly known to the *u*th UE [17].

### 2.3. Power Consumption and Energy Efficiency

Energy efficiency analysis requires careful modeling of the power consumption. In this paper, we focus on quantifying and minimizing the power consumption in the PAs. The power efficiency of the *b*th PA is defined as

(17) $$\eta_{b}=\frac{\rho_{\mathrm{tx}}^{\left(b\right)}}{\rho_{\mathrm{cons}}^{\left(b\right)}},$$

where $\rho_{\mathrm{tx}}^{\left(b\right)}$ denotes the output power, given by

(18) $$\rho_{\mathrm{tx}}^{\left(b\right)}=𝔼\left[|f_{b}\left(x_{b}\right){|}^{2}\right],$$

and $\rho_{\mathrm{cons}}^{\left(b\right)}$ represents the consumed power. Similarly to [5,6,7,8], we adopt the following simple yet accurate model for the power efficiency:

(19) $$\eta_{b}=\eta_{\max}^{\left(b\right)}\sqrt{\frac{\rho_{\mathrm{tx}}^{\left(b\right)}}{\rho_{\max}^{\left(b\right)}}}.$$

Here, $\rho_{\max}^{\left(b\right)}$ is the maximum output power of the *b*th PA, and $\eta_{\max}^{\left(b\right)}\in \left[0,1\right]$ denotes the maximum power efficiency obtained when $\rho_{\mathrm{tx}}^{\left(b\right)}=\rho_{\max}^{\left(b\right)}$. From (17) and (19), the consumed power at the *b*th PA can be expressed as

(20) $$\rho_{\mathrm{cons}}^{\left(b\right)}=\frac{1}{\eta_{\max}^{\left(b\right)}}\sqrt{\rho_{\mathrm{tx}}^{\left(b\right)}\rho_{\max}^{\left(b\right)}},$$

for b=1,⋯,B where $\rho_{\mathrm{tx}}^{\left(b\right)}\leq \rho_{\max}^{\left(b\right)}$. In other words, the consumed power is proportional to the square root of the output power, which was experimentally verified in various studies; see, e.g., Equation (6.93) in Reference [18].

The achievable sum rate in (15) and the power consumption model in (20) facilitate quantification of an energy efficiency metric, measured in bits per Joule, as

(21) $$\eta_{\mathrm{EE}}=\frac{WR_{\mathrm{sum}}\left(P\right)}{\rho_{\mathrm{cons}}^{\left(\mathrm{tot}\right)}\left(P\right)},$$

where *W* denotes the bandwidth and

(22) $$\rho_{\mathrm{cons}}^{\left(\mathrm{tot}\right)}\left(P\right)=\sum_{b=1}^{B}\rho_{\mathrm{cons}}^{\left(b\right)}\left(P\right)$$

is the total consumed power which can be evaluated using (20).

## 3. The Impact of SPAPR on Spectral and Energy Efficiency

**Figure 2:**
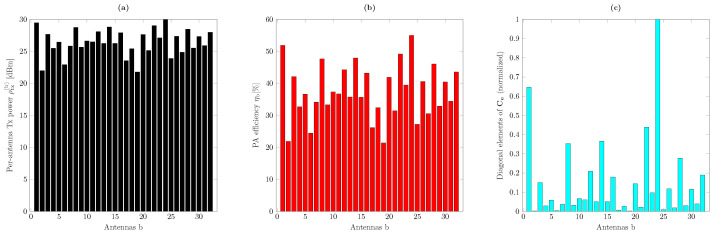
(**a**) Per-antenna transmit power values, (**b**) corresponding PA efficiencies, and (**c**) transmitted nonlinear distortion from different antennas. These results have been obtained by considering one realization of the geometric channel in (4) with B=32, U=4, and ZF precoding. The maximum output power is $\rho_{\max}^{\left(b\right)}=30$ dBm and the maximum power efficiency of the PAs is $\eta_{\max}^{\left(b\right)}=0.55$ for b=1,⋯,B. The nonlinearity characteristics of the PAs are as in (2) with K=1, $\beta_{1}^{\left(b\right)}=1$, and $\beta_{3}^{\left(b\right)}=-0.0426-0.0191j$.

As can be seen from the metrics formulated in Section 2.2 and Section 2.3, the choice of the precoder matrix P not only affects the spectral efficiency by controlling the useful signal power, multiuser interference, and distortion, but it also directly affects the power efficiency of the PAs and the overall energy efficiency of the system. In conventional precoding schemes, the structure of P is such that there is considerable variation in the average output power between antennas in the array at each coherence interval. An example of such power variation can be seen in Figure 2a, where we have plotted the per-antenna output power with ZF precoding and for one realization of the channel in (4) with a setup with B=32. Assuming $\rho_{\max}^{\left(b\right)}=30$ dBm and $\eta_{\max}^{\left(b\right)}=0.55$ for all b=1,⋯,B, the corresponding PA efficiencies are shown in Figure 2b. It can be seen that as a consequence of the power variation across the antennas, many of the PAs exhibit poor efficiency. Furthermore, as shown in Figure 2c, the contribution of these PAs to the nonlinear distortion is very different. More specifically, it can be seen that small backoffs can considerably reduce the amount of distortion. This is because the covariance of the distortion (12) is a function of $C_{x}⊙{\left|C_{x}\right|}^{2k}$, where $C_{x}=PP^{H}$, and thus the distortion decreases faster than linearly at reduced per-antenna powers.

Motivated by the observations made in Figure 2, in what follows, we seek precoding matrices that yield a favorable tradeoff between total power consumption and total radiated distortion. To this end, we propose precoding schemes with reduced power imbalance over different antennas. It is worth noting that our proposed solutions are different from precoding techniques with low peak-to-average power ratio (see, e.g., References [19,20]), which aim at reducing the power variation in the *temporal dimension*. They also differ from the solutions proposed in [21], where the power variation between antennas is reduced by precoding at the *symbol level*. Instead, we focus on block-level precoding schemes, which is the common approach in this context.

## 4. Precoding with Equal Antenna Powers

In this section, we present a family of linear precoding schemes with equal transmit powers from different antennas in the array. To this end, we propose exploiting the excess of spatial degrees of freedom in massive MIMO transmission to project part of the transmit power to the null space of the channel. In particular, the precoding matrices with equal antenna powers (EAP) can be generated as

(23) $$P_{\mathrm{EAP}}=\alpha \left(P_{\mathrm{conv}}+P_{⊥}Q\right),$$

where $P_{\mathrm{conv}}$ is the precoding matrix corresponding to a conventional precoding scheme, such as ZF and MRT, and $P_{⊥}$ is the orthogonal projection onto the null space of the channel matrix $H=\left[h_{1},\cdots ,h_{U}\right]$ given by

(24) $$P_{⊥}=I-H^{†}H,$$

where ${\left(\cdot \right)}^{†}$ stands for the pseudo-inverse operation and assuming that H has linearly independent rows, it is defined as $H^{†}=H^{H}{\left(HH^{H}\right)}^{-1}$. Moreover, Q is an arbitrary matrix, which will be used to guarantee the EAP condition, as explained below. Finally, α in (23) stands for the normalization factor, which is introduced such that the following two constraints are satisfied for different choices of precoding matrix:

1 The per-antenna power constraint (PAPC) given by
  (25) $$\rho_{\mathrm{tx}}^{\left(b\right)}=𝔼\left[|f_{b}\left(x_{b}\right){|}^{2}\right]\leq \rho_{\max}^{\left(b\right)},$$
  for b=1,⋯,B, where $x_{b}$ is the precoded signal input to the *b*th PA and can be obtained by multiplying the *b*th row of the precoding matrix P by the transmitted symbols s.
2 The total radiated power constraint (TRPC) given by
  (26) $$𝔼\left[{∥f(x)∥}^{2}\right]\leq \rho_{\mathrm{tot}}.$$

We now introduce a simple approach for finding Q such that the EAP transmission is realized. In particular, we start by generating a matrix $\tilde{P}$ without power variation across the antennas by normalizing the rows of $P_{\mathrm{conv}}$ such that

(27) $$𝔼\left[|f_{b}\left(x_{b}\right){|}^{2}\right]=\rho_{\max}^{\left(b\right)},$$

for all b=1,⋯,B. We then calculate the difference between $\tilde{P}$ and $P_{\mathrm{conv}}$ as

(28) $$\tilde{Q}=\tilde{P}-P_{\mathrm{conv}},$$

and find the matrix Q such that $\tilde{Q}=P_{⊥}Q$, namely, by calculating

(29) $$Q=P_{⊥}^{†}\tilde{Q}.$$

With a few iterations of (28) and (29), a precoder matrix is found that yields an almost zero power variation. The reason why we need multiple iterations is the numerical imprecision in the computation of $P_{⊥}P_{⊥}^{†}\tilde{Q}\approx \tilde{Q}$. In particular, the differences between $P_{⊥}P_{⊥}^{†}\tilde{Q}$ and $\tilde{Q}$ in the first iteration lead to some disturbances in the per-antenna powers which can be circumvented with some iterations of (28) and (29). A final step of normalization to satisfy the TRPC in (26) gives the final solution for $P_{\mathrm{EAP}}$.

The precoding scheme in (23) allows for preserving the structure of the conventional precoding matrix $P_{\mathrm{conv}}$. For example, if $P_{\mathrm{conv}}$ is substituted by the ZF precoder, the resulting precoding matrix for $P_{\mathrm{EAP}}$ also suppresses the multiuser interference as the remaining part of the signal is projected onto the null space of the channel.

**Figure 3:**
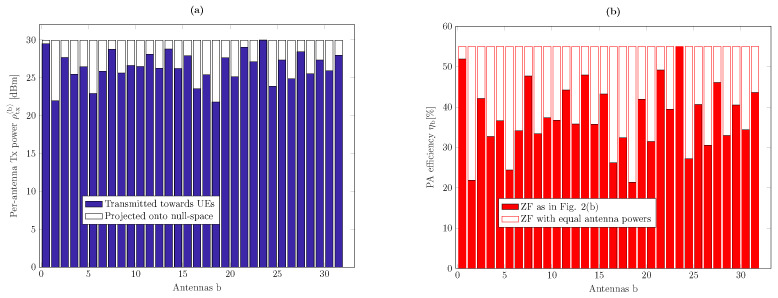
(**a**) Per-antenna transmit power values and (**b**) corresponding PA efficiencies for transmission with equal antenna powers versus conventional ZF for the same realization of the geometric channel as in Figure 2.

In Figure 3a, we plot the per-antenna power values for $P_{\mathrm{EAP}}$ obtained by replacing $P_{\mathrm{conv}}$ with the ZF precoding in Figure 2. The fractions of power transmitted in the direction of the UEs and the null space of the channel are shown in blue and white, respectively. The corresponding PA efficiencies are shown in Figure 3b and are compared with the PA efficiencies in conventional ZF precoding. The precoding scheme with EAP in (23) operates the PAs with their highest efficiency, namely with $\eta_{b}=0.55$ for all b=1,⋯,B in this particular example. Moreover, in precoding with EAP, unlike conventional ZF, where different PAs contribute very differently to the distortion (see Figure 2c), different PAs contribute almost equal amounts of nonlinear distortion, and therefore the total amount of distortion can be drastically reduced by small backoffs. However, this is achieved at the cost of increased total radiated power and by wasting some of this power by transmitting in the null space of the channel. Nevertheless, we show in Section 6 that precoding with EAP can lead to improved energy efficiency over conventional precoding schemes in certain output power ranges.

## 5. SPAPR-Controlled Distortion-Aware Precoding

Despite its attractive properties, the solution proposed in Section 4 can be significantly suboptimal in different scenarios, since a large amount of power is wasted when transmitting along the null space of the channel. Moreover, the conventional precoding schemes such as ZF do not take into account the effects of nonlinear distortion and thus may provide poor performance in the distortion-limited regimes. Motivated by these, in this section, we present a more advanced precoding technique that provides more flexible control over the power variation across antennas and further considers the effects of nonlinear distortion. In particular, we extend the solution in (23) by considering the following structure

(30) $$P_{\mathrm{SPDA}}=\alpha \left(P_{\mathrm{DA}}+\kappa P_{⊥}Q\right),$$

where the conventional precoder $P_{\mathrm{conv}}$ in (23) is replaced with a distortion-aware precoder $P_{\mathrm{DA}}$ and the parameter κ is introduced for a more flexible control of the power variation across the antennas. Next, we present a two-step optimization procedure for finding $P_{\mathrm{DA}}$ and κ. In the first step, similarly to Algorithm 1 in [10], we adopt a projected gradient ascent, to solve for

(31) $$\begin{array}{c} \begin{array}{cc} \underset{P_{\mathrm{DA}}\in C^{B\times U}}{\mathrm{maximize}}\;\; & \Gamma_{\mathrm{EE}}\left(P_{\mathrm{DA}}\right)=R_{\mathrm{sum}}\left(P_{\mathrm{DA}}\right)\;/\;\rho_{\mathrm{cons}}^{\left(\mathrm{tot}\right)}\left(P_{\mathrm{DA}}\right)\\ \mathrm{subject}\;\mathrm{to} & 𝔼\left[{∥f(x)∥}^{2}\right]\leq \rho_{\mathrm{tot}}\\ \mathrm{and} & \rho_{\min}^{\left(b\right)}\leq 𝔼\left[|f_{b}\left(x_{b}\right){|}^{2}\right]\leq \rho_{\max}^{\left(b\right)}\;\;\;\;\mathrm{for}\;\;b=1,\cdots ,B. \end{array} \end{array}$$

In particular, we seek a precoding matrix that maximizes energy efficiency under TRPC and PAPC. Here, $\rho_{\min}^{\left(b\right)}$ is introduced for two purposes. First, it prevents the algorithm from converging to a precoding matrix $P_{\mathrm{DA}}$ with very low $\rho_{\mathrm{cons}}^{\left(\mathrm{tot}\right)}$ and $R_{\mathrm{sum}}$ values. The second advantage of constraining the per-antenna power from below is that the optimization solutions inherently exhibit less power variation across the antennas in the array, which can be favorable in light of the analysis in Section 3 and Section 4.
**Algorithm 1** Steps for computing $P_{\mathrm{DA}}$ [10].**Inputs:** $h_{1},\cdots ,h_{U}$, $\beta_{1}^{\left(b\right)},\cdots ,\beta_{K}^{\left(b\right)}$, $\rho_{\min}^{\left(b\right)}$, $\rho_{\max}^{\left(b\right)}$, $\eta_{\max}^{\left(b\right)}$ for b=1,⋯,B, $\rho_{\mathrm{tot}}$, and $N_{0}$**Output:** $P_{\mathrm{DA}}$    *Initialization*: $\mu^{\left(0\right)}$ and $P^{\left(0\right)}$  1:$\Gamma_{\mathrm{EE}}^{\left(0\right)}\leftarrow MMR_{\mathrm{sum}}\left(P^{\left(0\right)}\right)\;/\;\rho_{\mathrm{cons}}^{\left(\mathrm{tot}\right)}\left(P^{\left(0\right)}\right)$  2:**for**i=1,⋯,I**do**  3: 
$\tilde{P}\leftarrow {\left[P^{\left(i-1\right)}\;+\;\mu^{\left(i-1\right)}\nabla_{P}\Gamma_{\mathrm{EE}}\left(P^{\left(i-1\right)}\right)\right]}^{+}$  4: 
${\tilde{\Gamma }}_{\mathrm{EE}}\leftarrow \Gamma_{\mathrm{EE}}\left(\tilde{P}\right)$  5: **if** ${\tilde{\Gamma }}_{\mathrm{EE}}>\Gamma_{\mathrm{EE}}^{\left(i-1\right)}$ **then**  6:  
$P^{\left(i\right)}\leftarrow \tilde{P}$, $\Gamma_{\mathrm{EE}}^{\left(i\right)}\leftarrow {\tilde{\Gamma }}_{\mathrm{EE}}$, and $\mu^{\left(i\right)}\leftarrow \mu^{\left(0\right)}$  7: **else**  8:  
$P^{\left(i\right)}\leftarrow P^{\left(i-1\right)}$, $\Gamma_{\mathrm{EE}}^{\left(i\right)}\leftarrow \Gamma_{\mathrm{EE}}^{\left(i-1\right)}$, and $\mu^{\left(i\right)}\leftarrow \frac{1}{2}\mu^{\left(i-1\right)}$  9: **end if**10:**end for**11:$P_{\mathrm{DA}}\leftarrow P^{\left(I\right)}$

To solve (31), similarly to Algorithm 1 in [10], we start with an initial precoding matrix $P^{\left(0\right)}$ and update it by taking steps along the steepest ascent direction of the objective function $\Gamma_{\mathrm{EE}}\left(P_{\mathrm{DA}}\right)=R_{\mathrm{sum}}\left(P_{\mathrm{DA}}\right)\;/\;\rho_{\mathrm{cons}}^{\left(\mathrm{tot}\right)}\left(P_{\mathrm{DA}}\right)$. The resulting precoding matrix is then normalized to ensure the feasibility of the solution. This procedure can be formulated as [22]

(32) $$\tilde{P}={\left[P^{\left(i-1\right)}+\mu^{\left(i-1\right)}\nabla_{P}\Gamma_{\mathrm{EE}}\left(P^{\left(i-1\right)}\right)\right]}^{+},$$

where i=1,⋯,I is the iteration index, *I* is the maximum number of iterations, $\mu^{\left(i\right)}$ is the step size of the *i*th iteration, and ${\left[\cdot \right]}^{+}$ denotes the normalization of the updated precoding matrix such that the two power constraints are satisfied. Once this normalization is applied, the objective function $\eta_{\mathrm{EE}}\left(\tilde{P}\right)=R_{\mathrm{sum}}\left(\tilde{P}\right)\;/\;\rho_{\mathrm{cons}}^{\left(\mathrm{tot}\right)}\left(\tilde{P}\right)$ is calculated, and if $\eta_{\mathrm{EE}}\left(\tilde{P}\right)>\eta_{\mathrm{EE}}\left(P^{\left(i-1\right)}\right)$, we update the precoding matrix to $P^{\left(i\right)}=\tilde{P}$ and reset the step size $\mu^{\left(i\right)}=\mu^{\left(0\right)}$. Otherwise, we do not update the precoding matrix, i.e., $P^{\left(i\right)}=P^{\left(i-1\right)}$, and decrease the step size $\mu^{\left(i\right)}=\frac{1}{2}\mu^{\left(i-1\right)}$. Finally, we choose $P_{\mathrm{DA}}=P^{\left(I\right)}$ as the output of the algorithm. The steps required for computing $P_{\mathrm{DA}}$ is summarized in Algorithm 1.

Now, for a fixed $P_{\mathrm{DA}}$, we obtain the matrix Q in (30) using a similar procedure as described in Section 4 and then find the optimal value for κ by conducting a simple line search over the interval [0,1] with the goal of maximizing $R_{\mathrm{sum}}\left(P_{\mathrm{SPDA}}\right)\;/\;\rho_{\mathrm{cons}}^{\left(\mathrm{tot}\right)}\left(P_{\mathrm{SPDA}}\right)$. The value of κ=1 corresponds to the special case with EAP precoding similar to the solution in (23). The optimization over κ provides an additional control on the SPAPR value, yielding a better tradeoff between the total consumed power and the amount of the radiated distortion.

## 6. Numerical Results

In this section, we perform numerical simulations to compare the performance of the solutions presented in Section 4 and Section 5 with the performance of conventional ZF precoding, assuming both perfect and imperfect CSI at the transmitter (CSIT). In our simulation setup, we assume that all base station antennas are equipped with identical PAs whose nonlinearity characteristics are modeled by (2) with K=1, $\beta_{1}^{\left(b\right)}=1$, and $\beta_{3}^{\left(b\right)}=-0.0426-0.0191j$. These coefficients have been obtained by linear regression on measurements over the class AB amplifier performed using [23]. The maximum output power at each antenna is $\rho_{\max}^{\left(b\right)}=30$ dBm, and the maximum power efficiency of the PAs is $\eta_{\max}^{\left(b\right)}=0.55$ for b=1,⋯,B [7]. The maximum total radiated power is $\rho_{\mathrm{tot}}=45$ dBm, and the variance of the AWGN is set to $N_{0}=-82$ dBm.

The channel coefficients are generated according to (4) and (5), where we set $N_{\mathrm{path}}=4$ and assume that the AoD $\psi_{u,\ell }$ is uniformly distributed over the interval $\left[0^{\circ },180^{\circ }\right)$. Moreover, we adopt the nLoS path loss model presented in [24] and, assuming that the system operates at a carrier frequency $f_{c}=28$ GHz, calculate the path loss for user *u* (at a distance of $d_{u}$ meters) using

(33) $$\gamma_{u}^{2}=-72-29.2\log_{10}\left(d_{u}\right)\;\;\;\;\;\left[\mathrm{dB}\right].$$

We further assume that the UEs are uniformly distributed in a disk-shaped area with the base station at its center. The minimum and maximum distances from the base station are set to $d_{\min}=5$ and $d_{\max}=35$ m, respectively. At this setting, the average path loss is approximately $\gamma_{\mathrm{avg}}^{2}=-108.5$ dB, which corresponds to a user at the distance of 17.8 m from the base station.

### 6.1. Performance Analysis under Perfect CSIT Assumption

**Figure 4:**
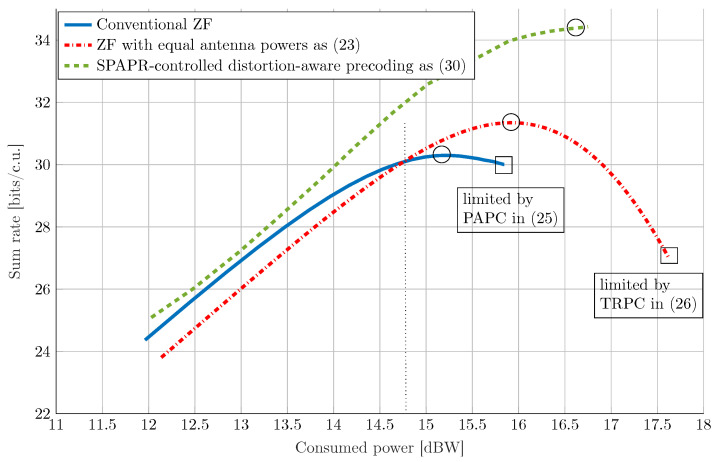
Energy efficiency comparison between $P_{\mathrm{SPDA}}$, $P_{\mathrm{EAP}}$, and conventional ZF precoding averaged over the realizations of the geometric channel (4) for a setup with B=32 antennas and U=4 UEs. The maximum output power at each PA is $\rho_{\max}^{\left(b\right)}=30$ dBm, and the maximum power efficiency is $\eta_{\max}^{\left(b\right)}=0.55$ for b=1,⋯,B. The nonlinearity characteristics of the PAs are as in (2) with K=1, $\beta_{1}^{\left(b\right)}=1$ and $\beta_{3}^{\left(b\right)}=-0.0426-0.0191j$ for b=1,⋯,B.

We first consider a setup with B=32 antennas and U=4 UEs. Assuming that the perfect CSI is available at the transmitter and UEs, we evaluate in Figure 4 the average sum rate (evaluated using (15)) versus the average total consumed power (computed using (22)) for three different precoding schemes, namely $P_{\mathrm{SPDA}}$ in (30), ZF with EAP in (23), and the conventional ZF by sweeping the total radiated power. It can be seen that the ZF with EAP has worse energy efficiency compared to the conventional ZF at the lower radiated power values. This is because in this regime, the performance degradation due to the power wasted in transmission along the null space outweighs the benefits that can be obtained by EAP transmission. However, at higher transmit powers, due to the operation of PAs with higher power efficiencies and due to the introduction of a smaller amount of nonlinear distortion, ZF with EAP outperforms the conventional ZF precoding, resulting in a higher maximum achievable sum rate (marked by circles in Figure 4). For instance, in Table 1, we compute the average received useful signal power $E\left[|h_{u}^{T}G\left(P\right)p_{u}{|}^{2}\right]$ and the average received nonlinear distortion power $E\left[h_{u}^{T}C_{e}\left(P\right)h_{u}^{*}\right]$ for a total average consumed power of 15.25 dBW (this is the point where ZF achieves its maximum achievable sum rate as depicted in Figure 4). It can be seen that ZF with EAP results in about 4.5 dB less distortion than ZF at the price of about 1 dB less average useful signal power, improving the overall signal-to-distortion ratio by about 3.5 dB. Figure 4 also shows that the SPAPR-controlled distortion-aware precoding yields a superior performance compared to ZF with EAP and conventional ZF. This improved performance is due to the consideration of the impact of nonlinear distortion in the precoder optimization procedure, as well as the mechanism for more flexible control of the SPAPR via optimization of the parameter κ. It is worth noting that the performance of the SPAPR-controlled distortion-aware precoding can be further improved by using a more sophisticated optimization to find the matrix Q instead of the procedure described in Section 4. However, this extension is beyond the scope of this paper and will be left to future work.

### 6.2. Performance Analysis under Imperfect CSIT Assumption

**Figure 5:**
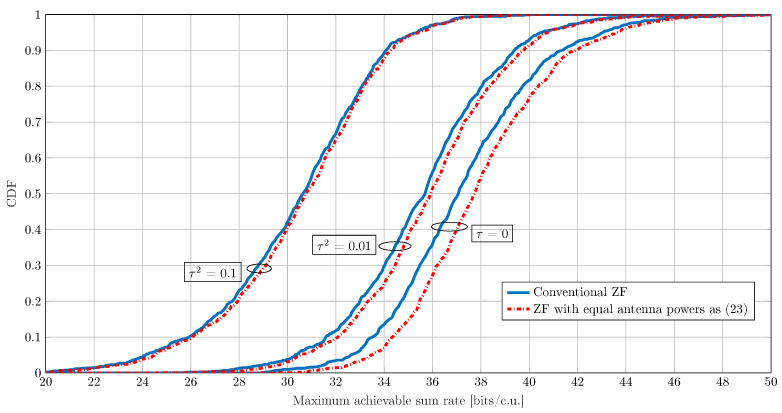
The CDF for the maximum achievable sum rate with $P_{\mathrm{EAP}}$ and conventional ZF precoding under different channel estimation qualities $\tau^{2}\in \left\{0,0.01,0.1\right\}$. These sum rate values are achieved over different realizations of the geometric channel (4) for an array with B=64 antennas and U=4 UEs. The maximum output power at each PA is $\rho_{\max}^{\left(b\right)}=30$ dBm, and the maximum power efficiency is $\eta_{\max}^{\left(b\right)}=0.55$ for b=1,⋯,B. The nonlinearity characteristics of the PAs are as in (2) with K=1, $\beta_{1}^{\left(b\right)}=1$, and $\beta_{3}^{\left(b\right)}=-0.0426-0.0191j$ for b=1,⋯,B.

The results in Figure 4 were obtained assuming perfect CSIT, but this assumption is not generally valid in practice. To investigate the impact of the channel estimation error on the performance of the proposed transmission schemes, we model the estimated CSIT with

(34) $${\hat{h}}_{u}=\sqrt{1-\tau^{2}}h_{u}+\tau v,$$

where the channel estimation error is modeled as an additive independent random error term. In particular, in (34), ${\hat{h}}_{u}$ and $h_{u}$ denote the estimated and the actual channel for the *u*th user, respectively. Moreover, τ∈[0,1] is a parameter reflecting the accuracy of the channel estimation, and the elements of the CSI error v are distributed according to $\mathrm{CN}\left(0,\sigma_{h_{u}}^{2}\right)$. In the following, we consider three different scenarios with different channel estimation accuracies. More precisely, we consider τ=0, which corresponds to the assumption of perfect CSIT, $\tau^{2}=0.01$, which is an example of a case with low to moderate channel estimation errors, and $\tau^{2}=0.1$, which reflects a scenario with poor channel estimation accuracy. For these scenarios, in Figure 5, we plot the cumulative distribution function (CDF) of the maximum achievable sum rate for ZF with EAP and the conventional ZF for a setup with B=64 antennas and U=4 UEs and over realizations of the geometric channel described in (4). As expected, both transmission schemes suffer from performance degradation in scenarios with imperfect CSIT, since precoding matrices computed using an erroneous channel lead to multiuser interference. More importantly, it can be seen that, while $P_{\mathrm{EAP}}$ can still provide a performance gain over the conventional ZF transmission in low to moderate channel estimation errors, this gain vanishes in the scenarios with large τ values (e.g., $\tau^{2}=0.1$). This is because with imperfect CSIT, the computed $P_{⊥}$ matrices in (23) are not perfectly orthogonal to the channel, leading to additional interference in the direction of the UEs. This additional interference can therefore negate the improvements that can be obtained by transmitting with the equal antenna powers. Extending the proposed solutions to achieve additional robustness to channel estimation errors is a subject of future studies.

## 7. Conclusions

We have studied the impact of per-antenna power distribution among different antennas in the array on the energy efficiency of a massive MIMO downlink system. In particular, we have shown that beneficial properties in terms of power efficiency and total radiated distortion can be achieved with a precoding matrix that operates all power amplifiers under the same backoff conditions. We have introduced a family of precoding matrices that can achieve these properties at the cost of some power wastage due to transmission in channel null space. Moreover, we have proposed a more advanced method to maximize energy efficiency by jointly optimizing the precoding matrix and the power variation across the antennas in the array. The performance gains that can be achieved with these precoding schemes over conventional zeroforcing precoding have been demonstrated using numerical experiments.

## Figures and Tables

**Table 1:** Resulting average received useful signal and nonlinear distortion power for conventional ZF and ZF with EAP for a total consumed power equal to $\rho_{\mathrm{cons}}^{\left(\mathrm{tot}\right)}=15.25$ dBW.

	Avg. Rx. Useful Sig. Power	Avg. Rx. Nonlinear Dist. Power
ZF	−85.62 dBW	−113.4 dBW
ZF with EAP	−86.36 dBW	−117.9 dBW

## Abbreviations

The following abbreviations are used in this manuscript:

AM/AM	amplitude-to-amplitude modulation
AM/PM	amplitude-to-phase modulation
AoD	angle of departure
AWGN	additive white Gaussian noise
CDF	cumulative distribution function
CSI	channel state information
CSIT	channel state information at the transmitter
EAP	equal antenna powers
MIMO	multiple-input-multiple-output
MISO	multiple-input-single-input
MRT	maximum-ratio transmission
PA	power amplifier
PAPC	per-antenna power constraint
RF	radio frequency
SINDR	signal-to-interference-noise-and-distortion ratio
SINR	signal-to-interference-plus-noise ratio
SNR	signal-to-noise ration
SPAPR	spatial peak-to-average power ratio
TRPC	total radiated power constraint
UE	user equipment
ULA	uniform linear array
ZF	zeroforcing
i.i.d.	independent and identically distributed
nLoS	non-line-of-sight
